# Prolonged cycling lowers subsequent running mechanical efficiency in collegiate triathletes

**DOI:** 10.1186/s13102-022-00543-w

**Published:** 2022-08-01

**Authors:** J. A. Stewart, E. K. Merritt, D. E. Lidstone, J. M. McBride, K. A. Zwetsloot

**Affiliations:** 1 Integrative Muscle Physiology Laboratory, Appalachian State University, ASU Box 32071, Boone, NC USA; 2 Biomechanics and Neuromuscular Laboratory, Appalachian State University, ASU Box 32071, Boone, NC USA; 3 Department of Health and Exercise Science, Appalachian State University, ASU Box 32071, Boone, NC 28608 USA; 4 Kinesiology Department, Southwestern University, 1001 E. University Ave., Georgetown, TX USA

**Keywords:** Running economy, Work, Energy expenditure, Muscle glycogen, Lactate

## Abstract

**Background:**

A significant challenge that non-elite collegiate triathletes encounter during competition is the decline in running performance immediately after cycling. Therefore, the purpose of this study was to determine if performing a 40-km bout of cycling immediately before running would negatively influence running economy and mechanical efficiency of running during simulated race conditions in collegiate triathletes.

**Methods:**

Eight competitive club-level collegiate triathletes randomly performed two trials: cycling for 40 km (Cycle-Run) or running for 5 km (Run–Run), immediately followed by a four-minute running economy and mechanical efficiency of running test at race pace on an instrumented treadmill. Blood lactate, respiratory exchange ratio, mechanical work, energy expenditure, and muscle glycogen were also measured during the four-minute running test.

**Results:**

Mechanical efficiency of running, but not running economy, was significantly lower in Cycle-Run, compared to Run–Run (42.1 ± 2.5% vs. 48.1 ± 2.5%, respectively; *p* = 0.027). Anaerobic energy expenditure was significantly higher in the Cycle-Run trial, compared to the Run–Run trial (16.3 ± 2.4 vs. 7.6 ± 1.1 kJ; *p* = 0.004); while net (151.0 ± 12.3 vs. 136.6 ± 9.6 kJ; *p* = 0.204) and aerobic energy expenditure (134.7 ± 12.3 vs. 129.1 ± 10.5 kJ; *p* = 0.549) were not statistically different between trials. Analysis of blood lactate, respiratory exchange ratio, mechanical work, and changes in muscle glycogen revealed no statistically significant differences between trials.

**Conclusions:**

These results suggest that mechanical efficiency of running, but not running economy, is decreased and anaerobic energy expenditure is increased when a 40-km bout of cycling is performed immediately before running in collegiate triathletes.

## Introduction

Triathletes are required to perform a variety of motor movements while maintaining a high level of intensity during the swim, cycle, and run phases of competition. Success in triathlons is dependent upon the athlete’s ability to perform these movements efficiently, and for prolonged durations. While elite triathletes are able to sustain a high running capacity after cycling [1], amateur triathletes can experience alterations in running performance after cycling [2, 3]. Although the underlying mechanisms responsible for these alterations in running performance after cycling have yet to be fully elucidated, it has been suggested that a combination of physiological and biomechanical factors play a role in the decline in running performance after cycling in a triathlon competition [4]

An important factor known to correlate with running speed in endurance events is running economy (RE). RE, the oxygen consumption at a given running pace, has been extensively utilized to assess running performance in various populations [5, 6] and RE is often used as an important measure of performance in trained runners [7]. In collegiate runners matched for aerobic capacity, RE was highly correlated with 10-km race times [5], thus emphasizing the importance of RE in running performance [6, 8]. However, the few studies that have assessed changes in RE immediately after cycling have provided conflicting results [9–11]. Bonacci et al. [11] demonstrated the change in a triathletes’ RE following a cycling bout is highly individual. Similarly, du Plessis et al. [12] also demonstrated the importance of assessing inter-individual responses to running after cycling. While oxygen consumption is an important component of running performance, numerous other variables, such as running mechanics, external mechanical work, fuel utilization, energy expenditure, and fatigue should also be considered when assessing running performance in competitive triathletes [4, 13, 14].

A more thorough assessment of performance than RE is mechanical efficiency during running (ME_R_). ME_R_ is the ratio of mechanical work to energy expenditure (both aerobic and anaerobic) [15–17]. McBride et al. [18] demonstrated that during a hopping protocol, competitive and recreational runners have similar external mechanical work values; however recreational runners have significantly higher aerobic and anaerobic energy expenditures, and thus lower mechanical efficiency during hopping, compared to competitive runners. Despite the importance of ME_R_ in athletic performance, to our knowledge only one other study has reported the inverse relationship between relative running intensity and mechanical work and energy expenditure, resulting in decreased ME_R_ [16]. Decreased ME_R_ after cycling may lead to declines in running performance in triathlon competition.

Furthermore, metabolic fatigue, induced through both aerobic and anaerobic pathways during strenuous bouts of cycling, could contribute to decreased muscle force and power observed after cycling [19]. Accumulation of metabolites, such as inorganic phosphate, has been shown to inhibit cross-bridge force production and would ultimately affect the force and power output of the muscle [20]. Performing a cycling task prior to running, as in a triathlon, the associated loss in muscle force and power could negatively influence both RE and ME_R_ [21]. Loss of muscle force and power may be reflected in observed decreases in muscle glycogen content [22], which has been shown to dramatically decrease after longer bouts of cycling [23].

It seems likely that alterations in RE and ME_R_, including variables such as oxygen consumption, mechanical work, energy expenditure, fatigue, and muscle glycogen, are contributors to this phenomenon. To date, no studies have measured both RE and ME_R_ during the cycle-to-run transition in simulated International/Olympic triathlon conditions. Therefore, the purpose of this investigation was to determine if performing a 40-km bout of cycling immediately before running would negatively influence RE and ME_R_ during simulated race conditions in collegiate triathletes. It was hypothesized that decreases in RE and ME_R_ after intense cycling would be associated with both a decrease in muscle glycogen and an increase in energy expenditure relative to external work.

## Materials and methods

### Study participants

Competitive, but non-elite club-level collegiate triathletes, 7 males and 1 female, age 18–30 years were recruited to participate in this study. All subjects were required to have a minimum of one-year experience competing in triathlon distances ranging from Olympic/International to Ironman. Participants were required to report to the lab for three separate visits, each separated by at least 48 h. Participants were informed about the benefits and risks associated with the study and completed the informed consent before participating. The Appalachian State University Institutional Review Board approved this study before any procedures began and all study procedures were conducted in accordance with the Declaration of Helsinki.

### Experimental design

The experimental design for this study was a counter-balanced crossover consisting of two randomized trials designed to simulate actual International/Olympic triathlon competition conditions. Each participant completed both trials at least 48 h apart. The Run–Run trial involved participants running a 5-km time trial (TT) at race pace, followed by a four-minute data collection run at race pace to measure running economy (RE), mechanical efficiency (ME_R_), blood lactate, respiratory exchange ratio (RER), mechanical work, energy expenditure (EE), and muscle glycogen. The Cycle-Run trial involved participants cycling a 40-km TT at race pace, followed by another four-minute data collection run. Participants were allowed to complete a sufficient, non-fatiguing warm up of their choice before each TT, but were instructed to keep their warm up routine the same for each trial.

### Procedures

During visit one, participants completed an informed consent, health screening questionnaire, anthropometric measures, and a VO_2_max test. The maximal graded exercise test was performed on the Bertec instrumented treadmill (Bertec; Columbus, OH) to assess individual maximal oxygen consumption. After obtaining baseline resting metabolic data (VO_2_, VCO_2_, RER, and V_E_; Parvo Medics 2400; Sandy, UT), participants were disconnected from the metabolic cart and asked to complete a 10-min warm up at a self-selected pace (no incline). Participants were then reconnected to the metabolic cart to obtain exercise metabolic data. Participants were instructed to begin the graded exercise test at their self-selected warm up pace and that the treadmill speed would increase 0.4 m•s^−1^ (no incline) for each successive stage until volitional exhaustion. Stages one through three were 4 min long, and every successive stage thereafter was 2 min long. Heart rate was recorded via a chest strap heart rate monitor connected to the metabolic cart (Polar Fit One; Kempele, Finland).

Upon arrival at the second visit, baseline resting blood lactate and muscle glycogen levels were recorded [23], and baseline metabolic data were obtained. Then participants randomly performed either the Run–Run trial or the Cycle-Run trial. All running was performed on the Bertec instrumented treadmill and cycling was performed on the triathlete’s own personal bicycle using a Computrainer® system (RacerMate; Seattle, WA). To ensure race pace consistency, heart rate was monitored throughout both the 5-km run TT and the 40-km cycle TT, but metabolic data were not collected. The 5-km TT was not intended to match the energy expenditure or work performed during the 40-km cycling TT, but rather to allow the triathletes a sufficiently high intensity, non-cycling workload prior to performing the four-minute data collection run. After completing the 5-km run TT (Run–Run trial) or the 40-km cycle TT (Cycle-Run trial), participants were allowed a 60- to 90-s transition (to simulate a triathlon transition period) to change shoes (for the Cycle-Run trial), record body mass, and don the face mask for metabolic measurements, before moving to the four-minute data collection run.

For the third visit, all procedures were repeated exactly the same as described above, however participants performed the trial not performed in the second visit. The second and third visits were completed at least 48 h apart.

#### Four-minute data collection run

The four-minute data collection run consisted of participants running on the Bertec instrumented treadmill at their competitive triathlon running race pace for four minutes. Data analysis for RE, ME_R_, RER, mechanical work, and EE occurred during the final two minutes of the four-minute data collection run to ensure steady state exercise. Race pace running speeds remained constant for each individual between trials and ranged between 3.33 and 4.44 m•s^−1^ (~ 75% of VO_2_ max) for all participants. RE was determined by measuring submaximal relative VO_2_ at each individual's self-selected race pace (between 3.33 and 4.44 m•s^−1^), as described previously [24]. Immediately after the four-minute data collection run, blood lactate was measured via finger prick using a Lactate Plus portable lactate analyzer (Nova Biomedical; Waltham, MA) to determine anaerobic energy expenditure.

#### Mechanical efficiency of running

Baseline/resting metabolic data were obtained before any activities were performed. During the resting data collection period, total O_2_ consumed in liters was recorded for two minutes to measure aerobic energy expenditure in kJ•L of O_2_^−1^ [17, 25, 26]. To assess ME_R_, forces from the footstrikes were utilized to calculate external mechanical work, and O_2_ consumption and RER were utilized to determine aerobic EE (EE_Aer_). EE was also calculated from changes in RER through a linear equation (kJ•L of O_2_^−1^ = 5.254•RER + 15.986) created by Zuntz and Schumburg [27]. Total O_2_ consumed for the data collection time period [∆time (min)•VO_2_ (in L•min^−1^)] was then multiplied by the kJ•L of O_2_^−1^ calculated from RER to provide energy produced. The sum of kJ of energy produced from the RER and total O_2_ consumed was considered baseline EE_Aer_. The baseline EE_Aer_ was subtracted from the kJ of energy produced from the RER and total O_2_ consumed during the exercise data collection period to measure changes in EE_Aer_. Anaerobic energy expenditure (EE_An_) was measured through changes in blood lactate. A resting lactate value was obtained during baseline metabolic data collection and subtracted from the lactate measured immediately after the four-minute data collection run. The change in lactate was then converted to O_2_ equivalents as 3 mL of O_2_•kg^−1^•mM^−1^ and multiplied by 21.1 kJ•L of O_2_^−1^ [28, 29].

To calculate external mechanical work (W_e_), vertical and horizontal center of mass (COM_b_) velocities (v) and displacements (h) were calculated from integration of acceleration values obtained via the force plates mounted within the Bertec treadmill [15]. The energy-time curve of the COM_b_ was provided by the summation of the potential (E*p* = m*g*h) and kinetic energies (E_k_ = ½mv^2^), where m is the mass of the subject and *g* is acceleration due to gravitational force (9.81 m•s^−2^). Thus, W_e_ (W_e_ = m*g*h + ½mv^2^) is represented by the incremental summation of this curve [30]. W_e_ was calculated as the positive work completed from each footstrike during the final two minutes of the four-minute data collection run to obtain steady state values [16, 17, 25, 26]. Every 15th footstrike was analyzed during the final two minutes, resulting in an average of 22 analyzed footstrikes at each participant’s race pace running velocity. Then, the total number of footstrikes analyzed was multiplied by the average positive work to obtain work values. ME_R_ was then calculated as the ratio between W_e_ and net (or total) energy expenditure (EE_n_ = EE_Aer_ + EE_An_), thus ME_R_ = W_e_/EE_n_, in accordance with previously published methods [16–18, 25].

#### Muscle glycogen assessment

Non-invasive muscle glycogen levels were obtained using the MuscleSound® ultrasound system, according to previous validation studies [23]. Participants were asked to lay supine while glycogen levels were measured in the rectus femoris muscle of the left leg in each subject before and after activity on visits two and three. A mark was made at half the distance from the patella to the inguinal crease to enable pre to post glycogen measurements at the same location for each trial and four images were obtained.

### Statistical analyses

All data are expressed as mean ± SEM. A Repeated Measures ANOVA was used to compare changes in blood lactate and muscle glycogen before and after running for the Run–Run and Cycle-Run trials. If significant F-ratios were found, within condition changes were compared post hoc using two-tailed t-tests with significance set after Bonferroni adjustment at *p* ≤ 0.0125. Paired sample t-tests were used to compare RE, ME_R_, absolute VO_2_, RER, EE, and mechanical work during running after the Run–Run and Cycle-Run trials. Effect sizes were computed for time × condition interactions using Cohen’s *d* and were interpreted such that 0.2, 0.5, and 0.8 were considered small, medium, and large effect sizes, respectively. Significance was set at *p* ≤ 0.05. All statistical analyses were performed using SPSS (IBM: Version 21.0. Armonk, NY). The data associated with this study are not publicly available, but are available from the corresponding author upon reasonable request.

## Results

**Fig. 1:**
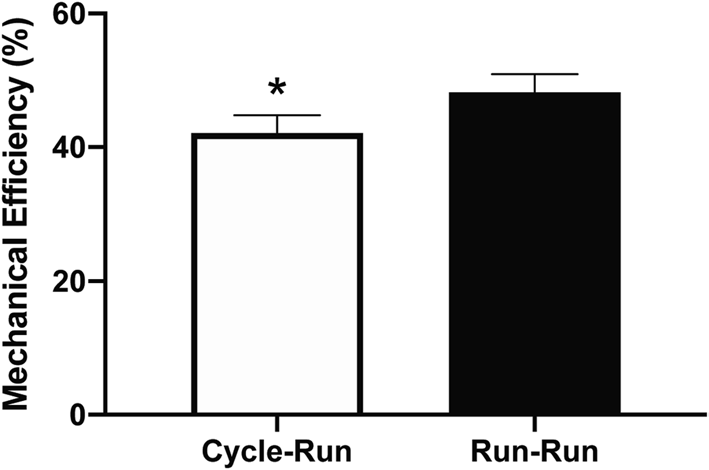
Mechanical efficiency of running (ME_R_) during the four-minute data collection run of the Cycle-Run and Run–Run trials. Data are expressed as mean ± SEM. * indicates significant difference between trials at *p* = 0.027

**Fig. 2:**
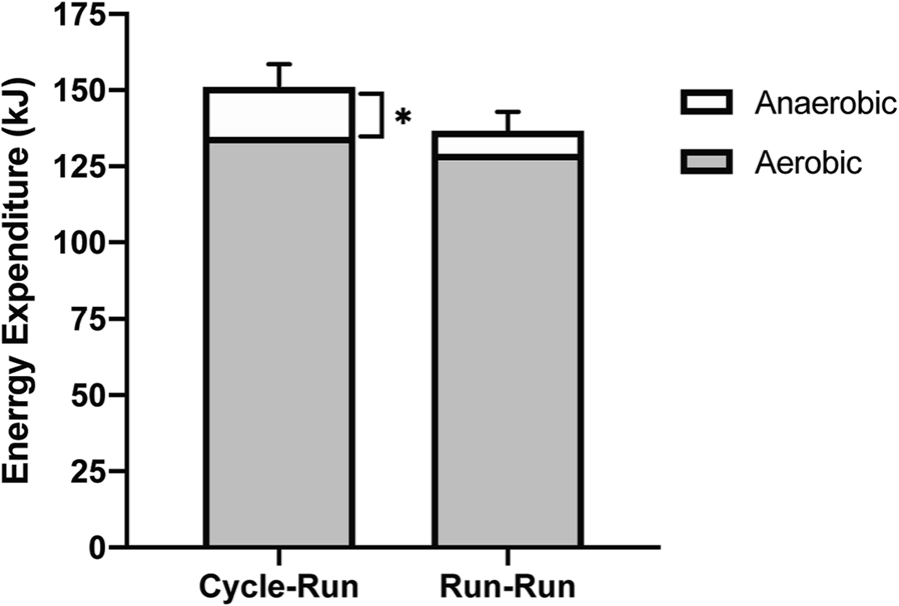
Energy expenditure during the four-minute data collection run of the Cycle-Run and Run–Run trials. Aerobic = aerobic energy expenditure; Anaerobic = anaerobic energy expenditure. Data are expressed as mean ± SEM. * indicates significant difference in anaerobic energy expenditure between trials at *p* = 0.004

**Fig. 3:**
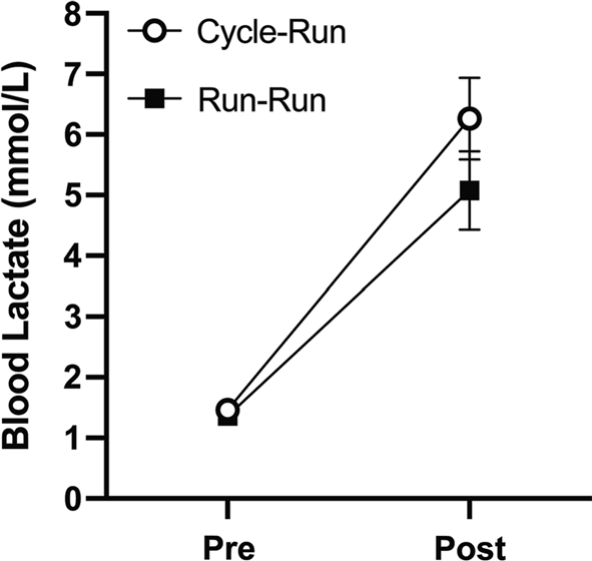
Change from rest in blood lactate concentrations immediately after the four-minute data collection run of the Cycle-Run and Run–Run trials. Pre = resting; Post = immediately after run. Data are expressed as mean ± SEM

Eight competitive collegiate triathletes (7 males and 1 female; age: 21.1 ± 0.5 yrs; height: 1.80 ± 0.03 m; mass: 74.0 ± 2.9 kg; VO_2peak_: 59.2 ± 2.5 mL•kg^−1^•min^−1^) completed this study. Mechanical efficiency of running during the four-minute data collection run was significantly lower in the Cycle-Run trial, compared to the Run–Run trial (42.1 ± 2.5% vs. 48.2 ± 2.5%, respectively; *p* = 0.027 [effect size 0.86]; Fig. 1). However, running economy, expressed as either a percentage of VO_2peak_ (74.8 ± 9.3 vs. 74.1 ± 7.8% VO_2peak_; *p* = 0.771 [effect size 0.08]) or as absolute VO_2_ (6.5 ± 1.3 vs. 6.4 ± 1.2 L•min^−1^; *p* = 0.804 [effect size 0.04]), did not differ between the Cycle-Run and the Run–Run trials, respectively (Table 1). While net EE (151.0 ± 12.3 vs. 136.6 ± 9.6 kJ; *p* = 0.204 [effect size 0.46]) and EE_Aer_ (134.7 ± 12.3 vs. 129.1 ± 10.5 kJ; *p* = 0.549 [effect size 0.18]) were not statistically different between the Cycle-Run and Run–Run trials, EE_An_ was significantly higher in the Cycle-Run trial, compared to the Run–Run trial (16.3 ± 2.4 vs. 7.6 ± 1.1 kJ; *p* = 0.004 [effect size 1.76]; Fig. 2). Analysis of blood lactate levels revealed no significant interaction between the two trials (*p* = 0.223), but there was a significant main effect of time, such that blood lactate levels increased similarly in both trials in response to exercise (p < 0.001 [effect size 0.68]). Although not statistically significant, the fold-change in lactate from rest appeared to be higher after the Cycle-Run trial, compared to after the Run–Run trial (3.82 ± 0.53-fold vs. 3.07 ± 0.69-fold, respectively; *p* = 0.179 [effect size = 0.43]; Fig. 3). There were also modest, non-significant differences in respiratory exchange ratio (0.96 ± 0.05 vs. 0.93 ± 0.02; *p* = 0.531 [effect size = 0.34]) and mechanical work (61.4 ± 2.0 vs. 64.0 ± 1.8 kJ; *p* = 0.137 [effect size = 0.50]) between the Cycle-Run and Run–Run trials, respectively (Table 1). There was also a similar, but not statistically significant decrease in muscle glycogen content after both the Cycle-Run and Run–Run trials, respectively (−16.0 ± 6.0% vs. −17.0 ± 9.0%; *p* = 0.860 [effect size 0.08]; Table 1). Average heart rate was 153 ± 13 bpm and time to completion was 75.6 ± 7.5 min for the 40-km cycling TT in the Cycle-Run trial; while average heart rate was 180 ± 8 bpm and time to completion was 22.1 ± 3.3 min for the 5-km running TT in the Run–Run trial.

**Table 1:** Performance and Physiological variables associated with Mechanical Efficiency and Running Economy measured during the four-minute data collection runs, data are reported as mean ± SEM

Variable	Cycle-Run	Run–Run	*p*-value
Mechanical efficiency (%)	42.1 ± 2.5	48.2 ± 2.5	0.027*
Relative RE (%VO_2peak_)	74.8 ± 9.3	74.1 ± 7.8	0.771
Absolute RE (L•min^−1^)	6.5 ± 1.3	6.4 ± 1.2	0.804
Mechanical work (kJ)	61.4 ± 2.0	64.0 ± 1.8	0.111
Net energy expenditure (kJ)	151.0 ± 12.3	136.6 ± 9.6	0.204
Aerobic energy expenditure (kJ)	134.7 ± 12.3	129.1 ± 10.5	0.549
Anaerobic energy expenditure (kJ)	16.3 ± 2.4	7.6 ± 1.1	0.004*
Blood lactate (fold-change)	3.82 ± 0.53	3.07 ± 0.69	0.179
RER (ratio)	0.96 ± 0.05	0.93 ± 0.02	0.531
Muscle glycogen (% change)	− 16.0 ± 6.0	− 17.0 ± 9.0	0.860

RE, running economy (expressed in terms of both relative and absolute VO_2_); RER, respiratory exchange ratio; kJ, kilojoules

*Significantly different between trials (*p* < 0.05)

## Discussion

This investigation aimed to determine if a 40-km bout of cycling performed immediately prior to running would negatively influence running economy (RE) and mechanical efficiency of running (ME_R_) in club-level collegiate triathletes. Here, we report the novel findings that ME_R_, but not RE, was significantly lower when a 40-km bout of cycling was performed prior to running, compared to prior running of 5 km. Furthermore, we reveal that anaerobic energy expenditure was significantly higher after 40 km of cycling, compared to after 5 km of running.

Mechanical efficiency is calculated as the ratio of work performed to EE [17], thus integrating both biomechanical and physiological parameters during muscular activities. In our triathletes, ME_R_ was significantly lower when a 40-km TT was performed before running, compared to running alone (42.1 ± 2.5% vs. 48.2 ± 2.5%; Fig. 1). Our calculated values for ME_R_ ranged from 38 to 58% for the collegiate triathletes in this study, which is comparable to other studies for running on treadmills at higher velocities [31]. While not statistically significant, slightly lower mechanical work performed, combined with slightly higher net energy expenditure in the Cycle-Run trial may have contributed to significant differences in ME_R_, despite running at the same intensity/speed in both trials. Gomes da Rosa et al. [32] reported that the metabolic cost of running was higher, but ME_R_ remained unchanged, when running was performed after cycling. However, their cycling and running stimuli were vastly different than the simulated triathlon race conditions performed by our subjects in the current study. All of their subjects cycled at a power intensity of 10% below recorded ventilatory threshold for 30 min, then immediately transitioned to running at 14 km•h^−1^ for 20 min.

Studies that include anaerobic energy expenditure in the assessment of mechanical efficiency provide more accurate representations of mechanical efficiency when running at higher velocities (> 3 m•s^−1^) [31], since anaerobic metabolism provides more ATP in these conditions, compared to slower running velocities. We observed that anaerobic energy expenditure was significantly higher in the Cycle-Run trial, compared to the Run–Run trial. The relative contributions of aerobic and anaerobic metabolism toward net energy expenditure during the four-minute data collection run were 89.2% and 10.8% in the Cycle-Run trial and 94.5% and 5.5% in the Run–Run trial, respectively (Fig. 2). These data suggest that the triathletes relied on anaerobic metabolism to a greater extent when a 40-km bout of cycling was performed prior to running, compared to prior running of 5 km. Furthermore, the modest elevation in lactate levels and RER during running after cycling suggest an increased reliance on carbohydrates for fuel, which might signify an increase in running intensity after cycling. The metabolic cost of running after cycling in triathletes has been investigated previously [2, 33]. Millet et al. reported no overall increase in the metabolic cost of running when mid-level triathletes performed a short (~ 3–6 min), exhaustive bout of cycling at 80% of maximal power, prior to running. However, this study reported only minor contributions from anaerobic energy expenditure (as indicated by changes in blood lactate) in running after exhaustive cycling [33]. Moreover, it is unlikely that triathletes would perform “exhaustive” cycling prior to the running phase of a triathlon competition. Findings from the current study are more aligned with those of Guezennec et al. [2] and Hausswirth et al. [3] who demonstrated the metabolic cost of running is higher after a prolonged bout of cycling, compared to after running alone, when running velocity is normalized. Other studies suggest that alterations in stride length, stride rate, and running mechanics can influence mechanical work and energy expenditure when cycling is performed before running, but we did not analyze these parameters [2, 3, 10, 32, 33]. Although we did not assess the effects of limb stiffness and repetitive stretch–shortening cycle movements on mechanical work, and thus efficiency, we feel it is important to recognize the significance of these variables in ME_R_.

While higher anaerobic energy expenditure (elevated blood lactate) and RER levels indicate greater reliance on glycolytic substrates, muscle glycogen depletion was not significantly different between trials. This suggests that muscle glycogen levels may not have been a major contributing factor for the lower ME_R_ observed after cycling as we had originally hypothesized. However, it is possible that the site of muscle glycogen assessment (rectus femoris muscle) was not wholly ideal for the type of exercise stimuli performed in this study. Perhaps, it would have been more ideal to assess muscle glycogen from multiple sites, including the vastus lateralis, rectus femoris, and gastrocnemius muscles to provide a better representation of total muscle glycogen levels, since muscle activation is different for various tasks [23].

### Practical applications

This study sought to identify the underlying biomechanical and physiological mechanisms involved with impairments in running mechanics or “heavy” legs experienced by many amateur triathletes when transitioning from the bike to run portions of the triathlon. Many triathletes experience this phenomenon and this study helps elucidate the possible reasons for why this happens. Our data suggest that triathletes transitioning from cycling to running experience a higher level of anaerobic metabolism, as indicated by elevated blood lactate levels and RER, compared to a previous, non-comparable bout of running. One possible technique to mitigate the decrement in running performance after cycling for triathletes competing or performing “brick” training, is to lower overall cycling intensity just slightly leading up to the transition to running (last 1–2 km of cycling) [4]. This may allow for anaerobic metabolism, and thus blood lactate levels, to subside enough to provide a smoother metabolic transition to the run portion of the triathlon.

## Conclusion

It is clear that running after cycling induces variable biomechanical and physiological responses, compared to previous running alone. While the mechanisms responsible for this phenomenon has not been fully elucidated, we observed significantly lower mechanical efficiency, but not running economy, and higher anaerobic energy expenditure (marked by modest elevations in blood lactate and RER levels) in running when a 40-km bout of cycling was performed prior to running, compared to prior running of 5 km. We believe this study provides insight for future investigations to examine different training interventions that aim to prevent the decline in mechanical efficiency during running after prolonged cycling in club-level collegiate triathletes. Furthermore, future directions for investigation might include assessing RE and MER before and after a 40-km bout of cycling.

## Data Availability

The datasets used and/or analyzed during this study are not publicly available, but are available from the corresponding author upon reasonable request.

## Abbreviations

Cycle-Run: Trial consisting of a 40-km cycling time trial prior to running
EE: Energy expenditure
EE_Aer_: Aerobic energy expenditure
EE_An_: Anaerobic energy expenditure
kJ: Kilojoules
ME_R_: Mechanical efficiency of running
Run–Run: Trial consisting of a 5-km running time trial prior to running
RE: Running economy
RER: Respiratory exchange ratio
TT: Time trial
V_E_: Ventilation expired
VCO_2_: Volume of carbon dioxide produced
VO_2_: Volume of oxygen consumed
W_e_: External mechanical work
